# The variation characteristics of fecal microbiota in remission UC patients with anxiety and depression

**DOI:** 10.3389/fmicb.2023.1237256

**Published:** 2023-09-07

**Authors:** Lingyun Xu, Yingchao Li, Yingli He

**Affiliations:** ^1^Department of Infectious Diseases, The First Affiliated Hospital of Xi'an Jiaotong University, Xi'an, China; ^2^Department of Gastroenterology, The First Affiliated Hospital of Xi'an Jiaotong University, Xi'an, China

**Keywords:** Ulcerative Colitis, psychological issues, fecal microbiota, probiotics, *Escherichia Shigella*

## Abstract

**Background:**

Ulcerative colitis (UC) patients with relapsed disease are most likely to suffer from anxiety and depression. Increasing data indicates that psychological issues can change the composition of intestinal flora. Thus, we aim to seek the variation of intestinal microbiota composition in remission UC patients with anxiety and depression in Northwest China.

**Results:**

In this study, 45 UC patients in remission were enrolled. The incidence of anxiety was 33.3%, and the prevalence of depression was 22.2%. There was no statistical difference in the alpha diversity of fecal microbiota, while beta diversity had a significant difference between the anxiety group and the non-anxiety group and the depression group and the non-depression group. Species composition analysis results showed that the ratio of *Bifidobacterium* and Lactobacilales significantly decreased. At the same time, the proportion of *Escherichia-Shigella* and *Proteus*_*mirabilis* increased in the anxiety group, and the ratio of *Faecalibacterium* and *Bifidobacterium* significantly decreased. In contrast, *Escherichia-Shigella* increased in the depression group at the gene levels.

**Conclusion:**

Anxiety and depression still exist in UC patients even in the remission period. We first identify that the proportion of probiotics decreases while the proportion of pathogens increases in UC patients with anxiety and depression. These findings may provide a new pathophysiological mechanism for the recurrence of disease caused by impaired psychological function and a new method for the treatment strategy of UC patients with psychological issues.

## Introduction

Inflammatory bowel diseases, Ulcerative Colitis (UC), and Crohn's disease are chronic idiopathic disorders causing inflammation of the gastrointestinal tract (Ng et al., 2017). The proportion of ulcerative colitis (UC) in IBD in China is significantly higher than in Western countries, which is a chronic inflammatory disease affecting the mucosa of the colon and rectum (Yang et al., 2022).

The most common psychological issues related to UC are anxiety and depression. The chronic inflammation associated with UC makes patients prone to psychological issues owing to their early diagnosis and lifelong symptoms (Neuendorf et al., 2016; Prendergast et al., 2022). There is a high prevalence of anxiety and depression in patients with UC, with up to one-third of patients experiencing anxiety symptoms and a quarter experiencing depression (Yu et al., 2023); female gender, lack of social support, and disease activity were associated with depression and anxiety in UC (Mikocka-Walus et al., 2016; Neuendorf et al., 2016; Williet and Sarter, 2017).

The relationship between UC and unhealthy psychological conditions seems bidirectional. The presence of abnormal anxiety scores at the baseline was associated with the later need for glucocorticosteroids or flare of UC activity and escalation of therapy in remission UC patients (Fairbrass et al., 2021), and depression at the baseline was related to an increased risk of aggressive UC at clinical follow-up (Kim et al., 2023). Despite this, the underlying mechanisms and association between psychological stress and IBD remain poorly understood.

The interaction between the brain and intestine mainly depends on the brain–gut axis. With the gradual understanding of the intestinal flora and its functions, the original “gut–brain axis” has been asserted to be the “gut–brain–bacteria axis” (Gracie et al., 2018; Basiji et al., 2023). Recent research studies revealed that intestinal flora can be affected by human psychological health (Wilkinson et al., 2019). Catecholamines can be released by sympathetic excitation by changing the colonization pattern of intestinal flora on the mucosal surface and leading to intestinal flora across the intestinal mucosa, which could mediate immune responses and cause intestinal inflammation (Mohajeri et al., 2018).

Taken together, anxiety and depression are common comorbidities in UC patients that can cause a flare of UC activity and escalation of therapy. The latest research finds that intestinal flora can be influenced by impaired psychological function. Thus, we aim to seek the variation of intestinal microbiota among UC patients with depression and anxiety, finding the potential pathophysiological mechanism for the recurrence of disease caused by impaired psychological conditions.

## Materials and methods

### Study population

From July 2021 to May 2022, this cross-sectional study was conducted at the First Affiliated Hospital of Xi'an Jiaotong University. All the UC subjects were in line with the Chinese consensus on diagnosis and treatment in IBD 2018 (Wu et al., 2018), and all the participants were in clinical remission (Mayo score ≤ 2 and no single subscore >1). Patients with other psychiatric disorders or who used antibiotics and probiotics in 1 month were excluded.

### Data collection

The information on the demographic and disease characteristics of UC patients was collected via an electronic medical record system. The patient's psychological condition was evaluated by accomplishing Hospital Anxiety and Depression Scale (HADS) (Zigmond and Snaith, 1983). Based on 16S rDNA sequencing technology, we analyzed alpha and beta diversities in fecal microbiota and the species composition differences of UC patients between positive and negative groups using bioinformatics.

### Microbial profiling

Fecal samples of all UC patients were collected and prepared. DNA was extracted from the fecal samples (HiPure Stool DNA Kits, D3141, Guangzhou Meiji Biotechnology Company, China), and the V3–V4 hypervariable region of bacterial 16S rRNA gene was sequenced (341F:CCTACGGGNGGCWGCAG 806R GGACTACHVGGGTATCTAAT). At a 98% similarity level, sequences were grouped into OTUs. In at least one sample, OTUs with relative abundances above 0.3% were retained.

### Questionnaires

The Hospital Anxiety and Depression Scale was used to assess patients' psychological conditions. The scale has two subscales, the Hospital Depression Scale (HDS) and the Hospital Anxiety Scale (HAS), both with a total score of 0–21. In previous studies, 8 is usually divided into the cutoff score of anxiety and depression.

### Statistical analysis

Alpha diversity refers to the Microbiota species diversity and evenness. A paired *t*-test of the Shannon Index was used to compare alpha diversity between different groups. A *P*-value of < 0.05 represented that the alpha diversity of different groups has statistical differences. Beta diversity analysis refers to the species differences among different bacterial communities. This study used principal coordinate analysis (PCA) that could find the differences in complex samples by reducing dimensions. We used Welch's test to analyze species composition differences, and a *P*-value of < 0.05 is the significant statistical threshold.

## Results

### Study population

A total of 45 UC patients were included in this study, and all of them had a median age of 49.8 ± 16.2 years. In terms of the duration of the disease, the participants had a median of 3.9 (IQR: 1.3–7.9) years; all of the participants were in remission period. In total, 31% of the patients had education of high school or below, and other demographic and disease characteristics of UC patients were summarized in Table 1.

**Table 1:** Sociodemographic characteristics of participants of the cohort.

**Characteristics**	**Total (*n =* 45)**	**Group-A (*n =* 15)**	**Group-NA (*n =* 30)**	**P-value**	**Group-D (*n =* 10)**	**Group-ND (*n =* 35)**	***P*-value**
**Gender**							
Male	29	8	21	0.12	5	24	0.08
Female	16	7	9		5	11	
**Age (years) 49.8** ±**16.2**							
18–30	5	1	4	0.77	0	5	0.19
30–60	33	10	23		8	25	
60–80	7	3	4		2	5	
**Education**							
Master's degree or above	17	6	11	0.26	4	13	0.23
Junior college	14	6	8		4	10	
Senior high school or below	14	3	11		2	12	
**Marital status**							
Unmarried	9	2	7	0.92	1	8	0.90
Married	36	13	23		9	27	
**Smoking status**							
Yes	13	4	9	0.65	3	10	0.58
No	32	11	21		7	25	
**Disease duration**							
Year, median	3.9	3.7	3.9	0.17	3.8	3.9	0.26
**Laboratory studies**							
WBC (× 109 /L)	6.56	6.59	6.55	0.33	6.54	6.57	0.56
ALB (g/L)	36.5	36.2	36.6	0.88	36.7	36.4	0.87
CRP (mg/L)	20.85	22.8	19.8	0.12	20.9	20.85	0.42
ESR (mm/h)	43	45	42	0.26	41	43.2	0.33
Calprotectin (ug/g)	45	48	43.5	0.18	44	45.2	0.63
**Medication**							
Mesalazine	18	5	13	0.65	4	14	0.82
Corticosteroid	7	2	5	0.08	1	6	0.17

Group-A indicates the anxiety group, Group-NA indicates the non-anxiety group. Group-D indicates the depression group, and Group-ND indicates the non-depression group.

^*^P < 0.05.

### Prevalence of anxiety and depression in remission UC patients

According to the findings of the HADS questionnaire, anxiety symptoms were found in 15 out of 45 UC patients, while depression symptoms were found in 10 out of 45 UC patients (Table 1). The prevalence of anxiety and depression is 33.3% and 22.2%, respectively. Further statistical analysis showed that gender, age, education level, marital status, disease duration, and medication did not differ between the groups (*P* > 0.05) (Table 1).

### Comparison of Alpha diversity between the groups

**Figure 1:**
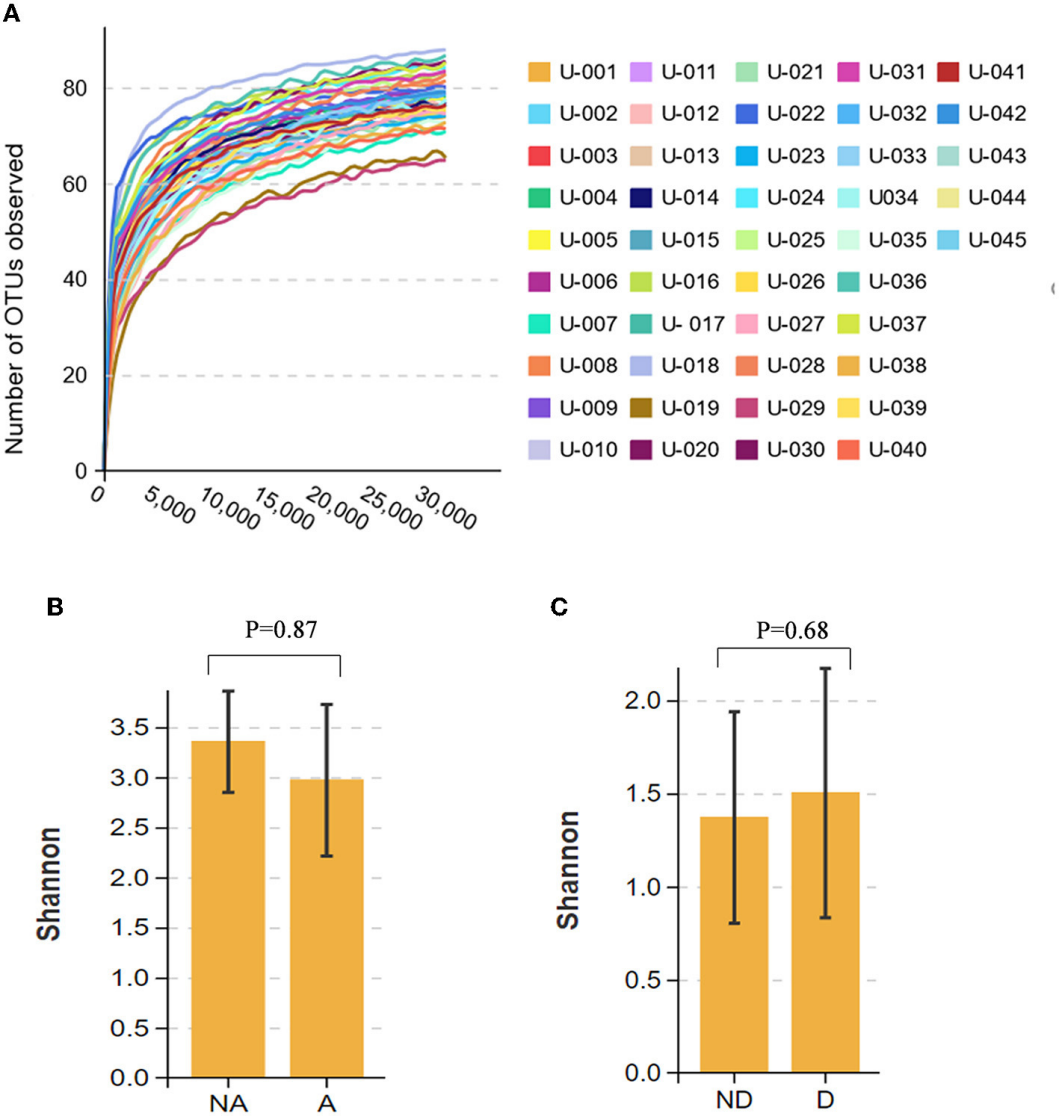
Comparison of alpha diversity between the groups. **(A)** The dilution curve of the fecal flora of all participants. **(B)** Shannon index comparison between the anxiety and the non-anxiety groups. **(C)** Shannon index comparison between the depression and non-depression groups. A indicates the anxiety group, and NA indicates the non-anxiety group. D indicates the depression group, and ND indicates the non-depression group.

The dilution curve of all fecal samples shows that the 30,000 tag sequence amounts that we provided were sufficient to cover most taxa and flora (Figure 1A). Alpha diversity represents the flora species richness and evenness, of which the Shannon index is the most representative index. Using the Shannon index, there were no significant differences in the species richness and diversity between the anxiety, non-anxiety, depression, and non-depression groups in remission UC (*P* > 0.05) (Figures 1B, C). The findings revealed that anxiety and depression did not affect the alpha diversity.

### Comparison of Beta diversity between the groups

**Figure 2:**
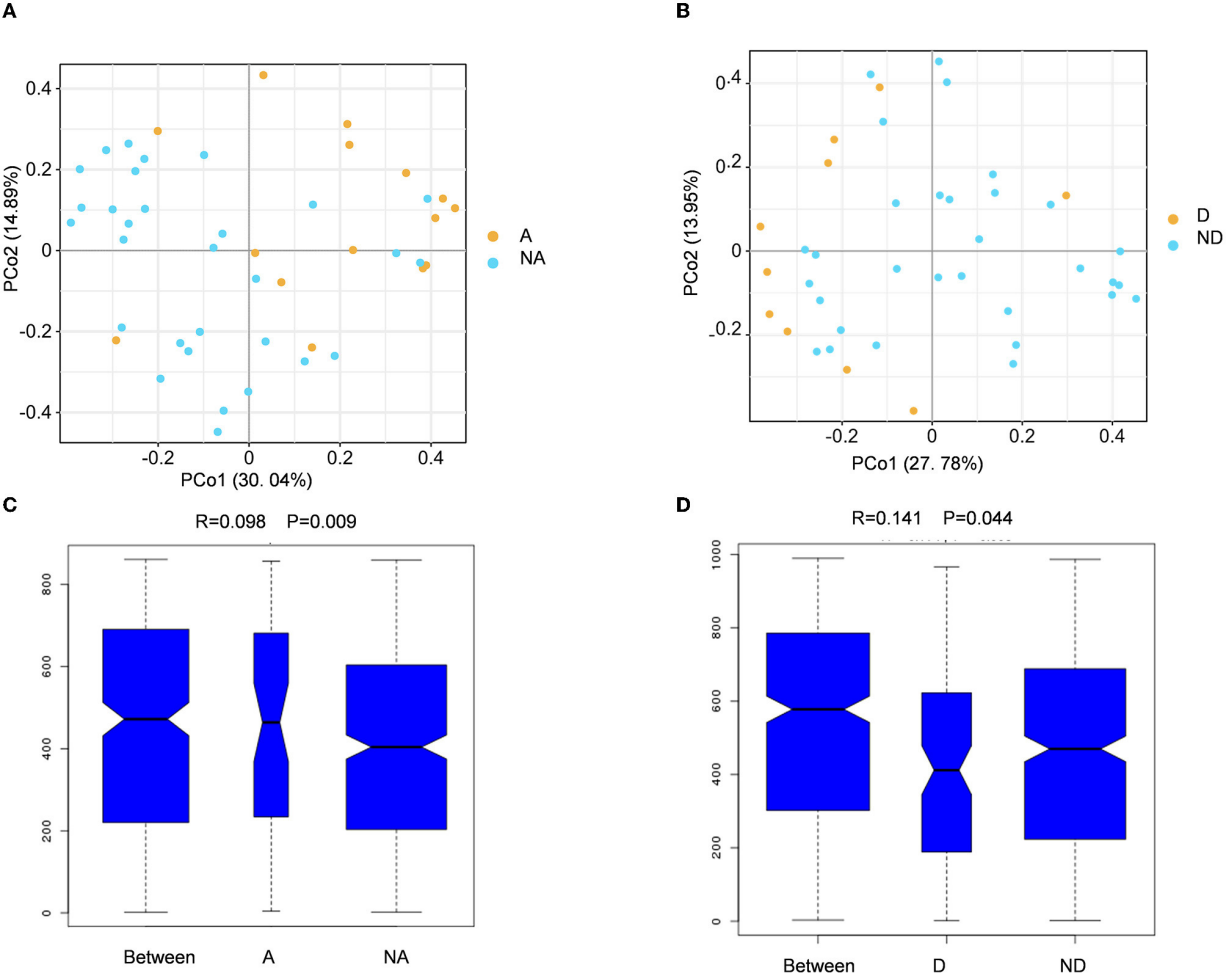
Fecal flora compositional differences associated with anxiety or depression. **(A, B)** The principal coordinate analysis (PCA) results between the anxiety group, non-anxiety group, depression group, and non-depression group. **(C, D)** Analysis of similarity between the groups. A indicates the anxiety group, and NA indicates the non-anxiety group. D shows the depression group. ND indicates the non-depression group.

Beta diversity refers to the comparison of species diversity among communities. The principal coordinate analysis (PCA) result showed that the yellow dots represented the anxiety group samples, and the blue dots represented the samples of the non-anxiety group. PCo1 contributes to the species variance of 30.04%, and PCo2 contributes to the species variance of 14.89% (Figure 2A). We found that all samples were divided into two clusters, indicating that the beta diversity was different between the anxiety and non-anxiety groups. The further analysis of similarity (ANOSIM) statistically indicated that anxiety changed the composition of the intestinal flora community in remission UC (R = 0.098, *P* = 0.009) (Figure 2C).

The principal coordinate analysis revealed that PC1 contributed 27.78% and the PC2 provided 13.95% to species variance between the depression group and the non-depression group when compared with the depression group (Figure 2B). The ANOSIM analysis revealed that the two groups have different bacterial community structures (R = 0.141, *P* = 0.044) (Figure 2D). There was a significant association between anxiety and depression and beta diversity of fecal microbiota in UC patients in remission.

### Species composition analysis with anxiety and depression

**Figure 3:**
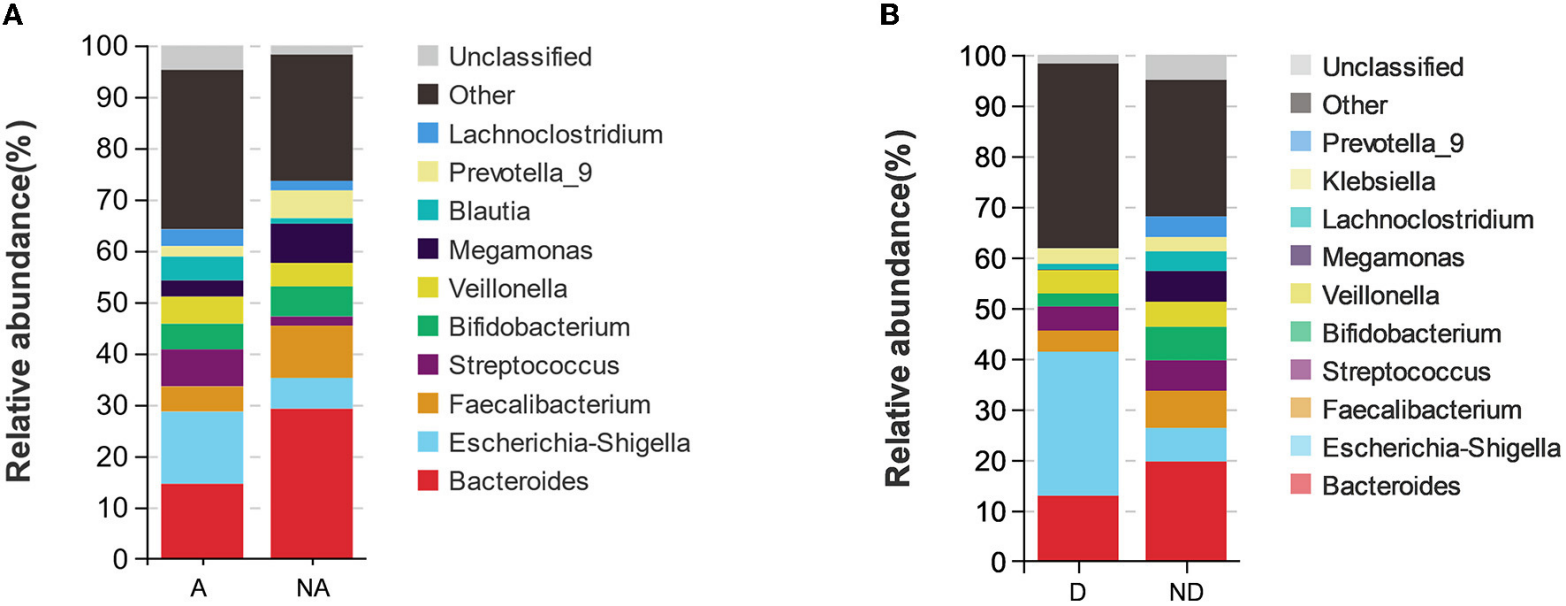
Species stacking chart between different groups. Species stacking chart shows the species proportions at the genus level in different groups in remission UC. **(A)** Species stacking chart between anxiety group and non-anxiety group. **(B)** Species stacking chart between depression group and non-depression group. A indicates the anxiety group, and NA indicates the non-anxiety group. D indicates the depression group, and ND indicates the non-depression group.

As shown in the species stacking chart (Figure 3A) and Table 2, the fecal flora with the largest composition ratio of the non-anxiety group was *Bacteroides, Faecalibacterium*, and *Megamonas*, while the main composition flora of the anxiety group was *Bacteroides, Escherichia-Shigella*, and *Megamonas*. Compared with the non-anxiety group, *Bifidobacterium* and *Lactobacillus* statistically decreased (*P* < 0.05), and *Escherichia-Shigella* and *Proteus*_*mirabilis* increased in the anxiety group (*P* < 0.05).

**Table 2:** Species abundance of fecal flora at the genus level in different groups in remission UC.

**Genus**	**Group-A**	**Group-NA**	***P*-value**	**Genus**	**Group-D**	**Group-ND**	***P*-value**
*Bacteroides*	14.09	28.47	0.11	*Bacteroides*	12.91	19.66	0.12
*Escherichia-Shigella*	12.50	5.43	0.04	*Escherichia-Shigella*	28.45	6.62	0.03
*Faecalibacterium*	4.39	9.50	0.43	*Faecalibacterium*	4.15	7.36	0.02
*Streptococcus*	6.57	1.67	0.73	*Streptococcus*	4.76	5.98	0.73
*Bifidobacterium*	4.61	5.54	0.01	*Bifidobacterium*	2.56	6.63	0.01
*Veillonella*	4.86	3.97	0.88	*Veillonella*	4.62	4.96	0.78
Lactobacilales	1.09	2.85	0.02	*Megamonas*	2.92	7.25	0.24
*Proteus mirabilis*	0.05	0.02	0.03	Lactobacilales	1.16	3.09	0.22

Group-A indicates the anxiety group, and Group-NA indicates the non-anxiety group. Group-D indicates the depression group, and Group-ND indicates the non-depression group.

According to the depression group, *Bacteroides, Escherichia, Shigella*, and *Streptococcus* had the largest fecal flora composition ratio. However, the proportion of fecal flora in the non-depression group was *Bacteroides, Faecalibacterium*, and *Escherichia-Shigella*. Compared with the non-depression group (Figure 3B), *Faecalibacterium* and *Bifidobacterium* were statistically decreased (*P* < 0.05), and *Escherichia-Shigella* was statistically increased (*P* < 0.05) in remission UC with depression.

### The variation characteristics of microbiota between patients only had anxiety/depression and suffered from both

**Figure 4:**
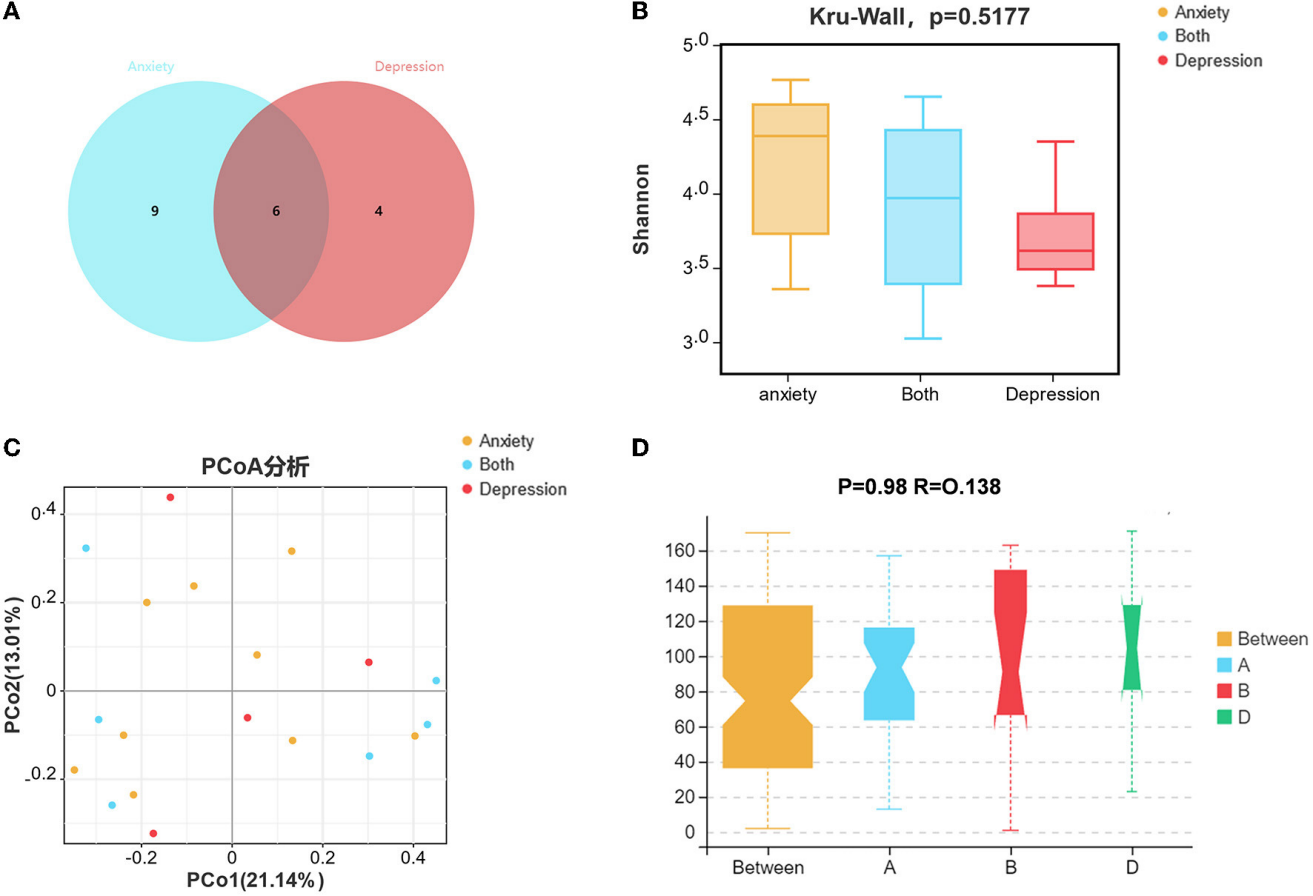
The variation characteristics of microbiota between patients who only had anxiety/depression symptoms and both suffered. **(A)** Venn diagram of patients only had anxiety/depression symptoms and both suffered. **(B)** Shannon index comparison between patients only had anxiety/depression symptoms and both suffered. **(C)** The principal coordinate analysis (PCA) results between the groups. **(D)** Analysis of Similarity between the groups.

Among 45 UC patients, six patients had both anxiety and depression symptoms (Figure 4A), nine patients had only anxiety symptoms, and four patients had only depression symptoms. The Wayne diagram is shown in Figure 4A. We analyzed the microbial communities of these three groups, but there were no statistically significant differences in alpha and beta diversity between the groups (Figures 4B–D).

### High abundance of *Escherichia-Shigella* may be associated with disease recurrence in remission UC patients

**Figure 5:**
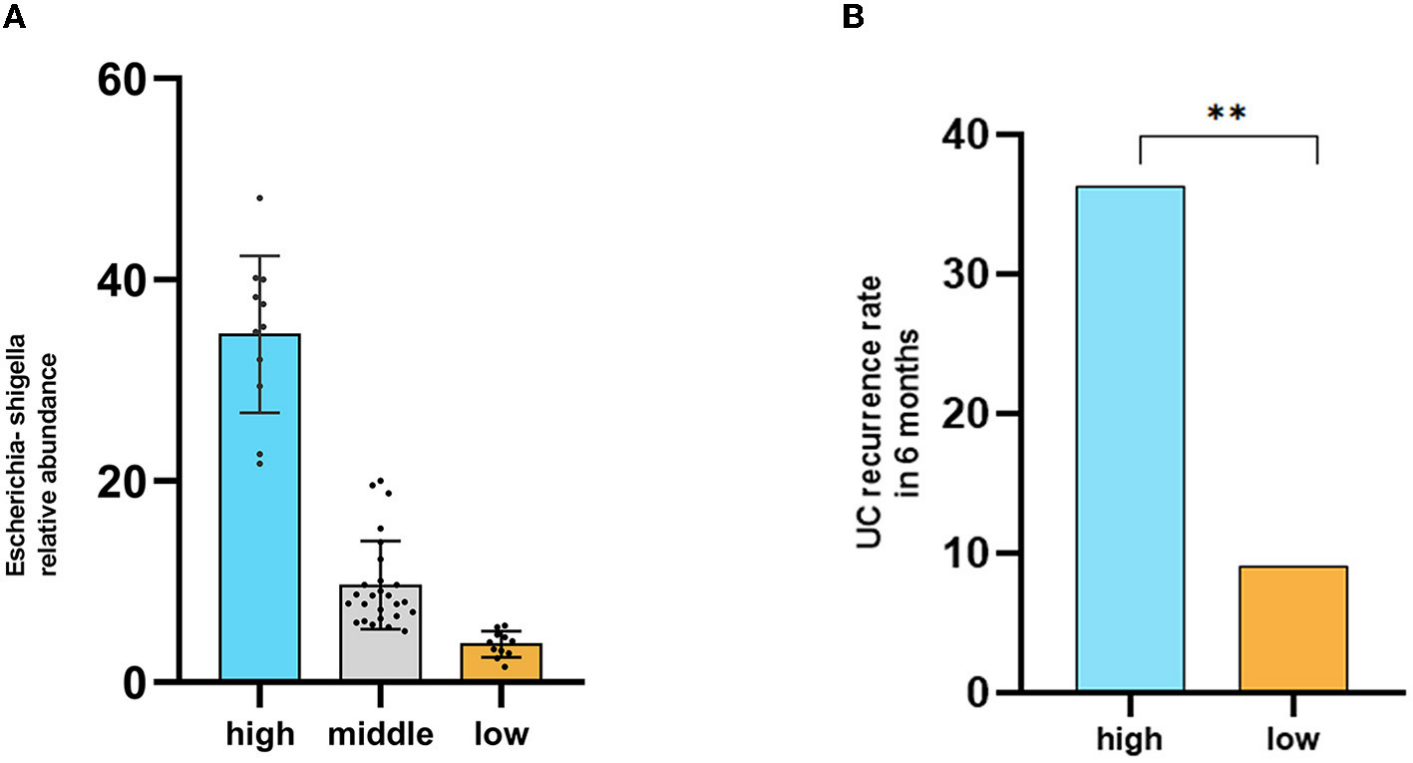
High abundance of *Escherichia-Shigella* may be associated with disease recurrence in remission UC patients. **(A)** Groups based on the abundance of *Escherichia-Shigella* using the quartile method. **(B)** Comparison of disease recurrence rates within 6 months. High indicates the high *Escherichia-Shigella* abundance group, middle indicates the middle *Escherichia-Shigella* abundance group, and low indicates the low *Escherichia-Shigella* abundance group.

Our research results found that *Escherichia-Shigella* significantly increased in both the anxiety and depression groups. Due to the lack of a gold standard, we used the quartile method to classify the relative abundance of *Escherichia-Shigella* among all participants, mainly including the high content group (*n* = 11), middle content group (*n* = 23), and low content group (*n* = 11). The relative abundance of *Escherichia-Shigella* in each group of patients is shown in Figure 5A. We followed up the disease recurrence rates of high and low content groups for 6 months which were 36.3% and 9% (Figure 5B) (chi-square test, *P* < 0.05).

## Discussion

We carried out a cross-sectional study in UC that aims to examine the incidence of anxiety and depression in disease remission and the variation of fecal microbiota in anxious and depressed UC patients in remission.

Our study results showed that even in the remission period, the prevalence of anxiety was 33.3%, and depression was 22.2%, which was more than twice the incidence of anxiety and depression in healthy individuals. Anxiety and depression are more prevalent in UC patients with remission due to repeated illnesses, hormones, economic hardship, and other factors (Sidebottom et al., 2021). In some clinical studies, anxiety and depression trigger disease recurrence, surgery, or hospitalization (Regueiro et al., 2016). Therefore, doctors should be careful regarding anxiety and depression in UC patients, even in the remission period (Gao, 2021; Jordi et al., 2022).

Alpha diversity was not significantly different between remission UC patients with anxiety/depression (UCA/UCD) and UC without anxiety/depression (UCNA/UCND), but beta diversity was significantly different (*P* < 0.05). Consistent with our research results, the latest research suggests that psychological stress could change the composition of intestinal microbiota and promote intestinal inflammation (Bailey et al., 2011; Li, 2019). Increasing attention has been placed on the brain's and microbiota's interactive regulatory mechanism during disease progression (Morais et al., 2021).

We further found that the abundance of harmful or pathogenic bacteria increased, and the proportion of probiotics decreased in UCA/UCD compared with UCNA/UCND in the remission period. In previous animal experiments, acute and chronic stress could both promote the expansion of pathogens and activate the mucosal immune response, and antibiotic treatment eliminated the intestinal microbiota factor and the difference between the stressed and non-stressed groups (Ge et al., 2022). Microbial metabolites also participate in the pathogenesis of colitis. Short-chain fatty acids (SCFAs), one of the important metabolites of intestinal flora, play a major role in several physiological processes, including maintaining the intestinal barrier, inhibiting opportunistic intestinal pathogen colonization, and regulating the Treg cell balance (Bravo et al., 2011). In addition, the components of some harmful bacteria, such as LPS and some types of proteases, can destroy the production of tight junction proteins, thus damaging the intestinal barrier and causing intestinal inflammation (Zou et al., 2022).

We found that the abundance of *Bifidobacterium* and *Lactobacillus* decreased, while the abundance of *Escherichia-Shigella* and *Proteus*_*mirabilis* increased in the anxiety group. The abundance of *Bifidobacterium* and *Faecalibacterium* decreased in the depression group, while the abundance of *Shigella* increased at the genus level. In the study by Bravo, *Lactobacillus* was shown to be able to improve anxiety and depression-related behavior in BALB/c mice; both *Bifidobacterium* and *Lactobacillus* have been proven to improve anxiety and depression (Sudo et al., 2004). Anxiety symptom of animals supplemented with rhamnose-Lactobacillus was improved and accompanied by changing the expression of the γ-aminobutyric acid receptor (Štofilová and Kvaková, 2022). Human studies also showed that intestinal microbes played a role in regulating depression and anxiety. The mental health of petrochemical workers improved for 6 weeks after eating probiotic yogurt or multi-species probiotic capsules, improving their mental health for 6 weeks (Kim et al., 2019). In a recent randomized double-blind controlled trial in central Iran, the clinical symptoms of patients with severe depression were improved compared with those of the placebo group, after 8 weeks of treatment by probiotics (including *Lactobacillus acidophilus, Lactobacillus lactis*, and *Bifidobacterium)* (Mohammadi et al., 2015; Akkasheh et al., 2016). Probiotics could, therefore, be used to improve the symptoms of UC patients in remission with anxiety and depression.

Probiotic treatment currently includes probiotics and fecal microbiota transplantation (FMT) (Kilinçarslan and Evrensel, 2020). FMT has become an attractive therapeutic strategy. Some studies have shown that the severity of anxiety and depression in IBD patients is improved after FMT (Jang et al., 2019; Qiu et al., 2023). Based on those studies, we believe that for UCA/UCD patients in the remission stage, oral probiotics can be added in order to improve symptoms and disease recurrence. In addition to traditional psychotherapy and drug therapy, FMT may become an important method to improve the mental state of UC patients with severe anxiety and depression.

Interestingly, we followed up the disease recurrence rates of high and low *Escherichia-Shigella* content groups for 6 months were 36.3% and 9%. Consistent with our results, *Escherichia coli* is closely related to localization and recurrence of UC. As an antibacterial therapy, phage therapy has the advantage of precise targeting compared with antibiotics and can be used to regulate intestinal flora and kill multiple types of drug-resistant bacteria (Zheng et al., 2022).

However, our study is a single-center study with insufficient subjects to represent all UC patients. The sample size should be further expanded, and the conclusion should be verified in multi-centers.

## Conclusion

This study enrolled 45 UC patients in remission. The majority of UC patients remain anxious and depressed even in the remission period. In this study, we found that probiotics decreased, and conditional pathogenic bacteria were more prevalent in UC patients in remission with anxiety/depression compared with UC in the remission stage without anxiety/depression. Therefore, supplementing probiotics may be an important method to prevent the recurrence of disease caused by impaired psychological function.

## Data availability statement

The raw data supporting the conclusions of this article will be made available by the authors, without undue reservation.

## Ethics statement

The studies involving humans were approved by the First Affiliated Hospital of Xi'an Jiaotong University. The studies were conducted in accordance with the local legislation and institutional requirements. Written informed consent for participation in this study was provided by the participants' legal guardians/next of kin.

## Author contributions

YL designed the study. LX drafted the manuscript and performed the experiments. YH revised the manuscript. All authors contributed to the article and approved the submitted version.
